# Digital restoration of colour cinematic films using imaging spectroscopy and machine learning

**DOI:** 10.1038/s41598-022-25248-5

**Published:** 2022-12-20

**Authors:** L. Liu, E. Catelli, A. Katsaggelos, G. Sciutto, R. Mazzeo, M. Milanic, J. Stergar, S. Prati, M. Walton

**Affiliations:** 1 Department of Informatics-Science and Engineering, University of Bologna, Mura Anteo Zamboni, 7, Bologna, Italy; 2 Department of Chemistry “G. Ciamician”, University of Bologna, Via Guaccimanni, 42, 48121 Ravenna, Italy; 3 Department of Electrical Engineering and Computer Science, Northwestern University, 3270, Evanston, IL USA; 4 Faculty of Mathematics and Physics, University of Ljubljana, Jadranska Cesta 19, 1000 Ljubljana, Slovenia; 5 “Jožef Stefan” Institute, Jamova Cesta 39, 1000 Ljubljana, Slovenia; 6 Department of Conservation and Research, M+ Museum, 38 Museum Drive, West Kowloon Cultural District, Hong Kong

**Keywords:** Chemistry, Analytical chemistry, Imaging studies

## Abstract

Digital restoration is a rapidly growing methodology within the field of heritage conservation, especially for early cinematic films which have intrinsically unstable dye colourants that suffer from irreversible colour fading. Although numerous techniques to restore film digitally have emerged recently, complex degradation remains a challenging problem. This paper proposes a novel vector quantization (VQ) algorithm for restoring movie frames based on the acquisition of spectroscopic data with a custom-made push-broom VNIR hyperspectral camera (380–780 nm). The VQ algorithm utilizes what we call a multi-codebook that correlates degraded areas with corresponding non-degraded ones selected from reference frames. The spectral-codebook was compared with a professional commercially available film restoration software (DaVinci Resolve 17) tested both on RGB and on hyperspectral providing better results in terms of colour reconstruction.

## Introduction

Digital restoration is a rapidly growing methodology in cultural heritage whereby images of art objects are computationally manipulated to visualize their original appearance or reveal hidden information without actual physical intervention^1–4^. Digital restoration is increasingly playing a role in interpreting and displaying an artwork when it is severely damaged^5,6^ or when it has been stripped of historically significant information^7^.

As has been recognized by UNESCO since 1980, moving images are a fundamental part of the world's Cultural Heritage^8^. Throughout the twentieth century, films were coloured with light and heat-sensitive dyes incorporated into the emulsion layers. Today, these films often exhibit colour degradation, fading, colour loss, bleaching, and colour change^8^, thus necessitating their digital restoration^9–11^. For motion pictures, the film is commonly restored by scanning using an RGB scanner and manually processed with dedicated software, such as *Photoworks Photo Editor 2021*^12^, *DaVinci Resolve 17* by Black Magic^13^, and *Paintshop Pro* by Corel^14^, to re-balance the colour and adjust the colour saturation and contrast^10^. Conventional digital restoration is laborious, with the resulting appearance reliant upon the restorers' skills and judgments about what looks appropriate.

This study proposes a machine learning algorithm that avoids subjective choices in restoring differentially faded film. As described in more detail below, a vector quantization algorithm is proposed that exploits a sparse representation of spectral reflectance data obtained from degraded and non-degraded films. After registration of representative degraded and non-degraded frames, a joint dictionary is learned from these data sets, which calculates a restored representation for the entire film. Spectral data were first processed using a simple codebook approach and further improved by a multi-codebook method capable of restoring frames with different degradation effects. The method proposed here provides more accurate results than those obtained with the currently available restoration software.

### Previous work

In response to these subjective approaches, several algorithms have been developed to automatically restore digitalized films with minimal intervention^9,15^. Several of these techniques successfully detect scratches or lacunae, and this missing content is in-painted using standard techniques^4,16–18^. However, for faded colour, most existing models assume homogeneous reduction in colour and hue across the image frame. Only one deep learning algorithm, based on latent space translation, trains with paired synthetic data^19^ to compensate for uniform fading. For more severe and inhomogeneous colour loss, the algorithm is prone to failure. Other CNN algorithms focusing on colorization of black and white films^20–23^ rely on synthetic training data sets that have the same limitation when it comes to uneven fading. In historical films, the degradation of colour usually varies across and within each frame, so restoration models trained using many homogeneous synthetic images may impose inaccuracies or even colour distortions. Another approach, based on what is known as the *Automatic Colour Equalization Model*^11^, imitates the mechanics of the human visual system, optimizing colour contrast, saturation, and balance according to human perception and aesthetics rather than restoring the film to its original appearance. Such methods are generally ineffective when attempting to restore artworks to a historically accurate state, as is the central requirement in the cultural heritage sector.

Practical restoration of differentially degraded colour film thus remains an unsettled problem. Here we propose advanced tools, such as spectral imaging, to face the challenges imposed by the complexity of colour degradation in historical films. Hyperspectral imaging has been increasingly applied to the analysis and conservation of important artefacts^24–26^. The fine spectral resolution afforded by optical reflection spectroscopy, down to nanometre resolution, enables the capture of degradation phenomena of film at high spatial and spectral resolution, which is otherwise hard to identify with the conventional RGB captures. By combining spectral imaging with advanced machine learning algorithms, the limitations of using synthetic data alone is overcome, given the large amount of spectral data that may serve as the input to the algorithm. In addition, machine learning also handles the challenge of processing large amounts of data which is often a major concern in cultural heritage applications. Such methodology has already been reported in the study of illuminated manuscripts where hyperspectral imaging and a deep neural network were combined to perform the spectral unmixing and quantitative estimation of pigment concentrations^27^. Another important work on the degraded medieval manuscript^28^ proposed a codebook algorithm to fuse the hyperspectral data and XRF data that successfully revealed the hidden content through the correlated spectral mapping. Although no application of this approach has been reported on film restoration, those research projects open the door for a novel solution to the colour degradation problem in damaged historical films.

## Materials and methods

### Materials

**Figure 1:**
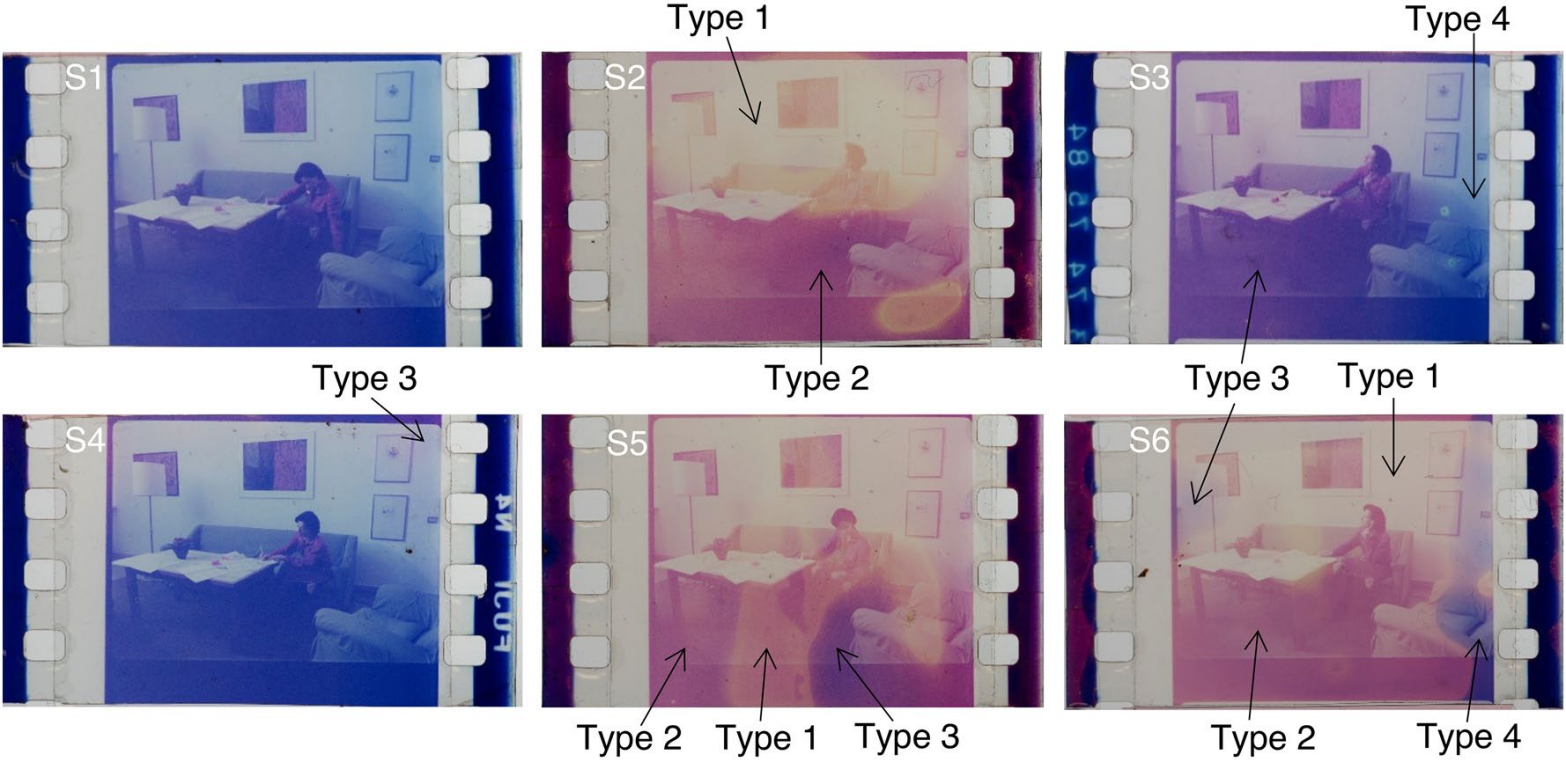
Optical RGB images captured by Canon EOS 5D Mark IV camera of the film samples (S1–S6) considered in this work.

Six positive frames (Fig. 1) belonging to the same scene but with different fading effects were provided by L'immagine Ritrovata Film Restoration Laboratory (Cinematique of Bologna). This type of colour film has a trichromatic structure in which the yellow, magenta, and cyan dyes are in their own layers in the emulsion and degrade separately at variable rates over time. A more detailed description of these samples is provided in the supplementary information (SI, paragraph A).

The best-preserved film S1 is regarded as "the good" reference to form the codebook. From the degraded frames, four different fading effects are recognizable, as indicated in Fig. 1 (type 1–4):

1. A yellowish hue is formed due to the decomposition of cyan and magenta dyes (yellowish upper part in S2).
2. A pinkish hue in S5 and S6 is due to the degradation of the cyan dye.
3. Purplish hue on the left part of S3 and upper part of S4, probably due to a very mild degradation that leaves an amount of the cyan dye and keeps most of the colour density.
4. Bluish strips on the right of S3 and S6, as well as sample S1, are considered un-faded parts that preserve most of the dyes.

Sample S6, containing pixels presenting all the four described fading effects, provides the richest information on the fading and is selected as the fade reference.

### Data acquisition methods

The frames were scanned with a custom-made VNIR push-broom hyperspectral camera^29^, specifically built to have high spatial and spectral resolutions. Spectral images were acquired in a reflectance geometry with a broadband LED light source covering the spectral range from 380 to 1000 nm^29^. The light source was configured to illuminate the whole sample homogeneously from two sides while using a combined diffusor/polarizer (Bolder Vision Optik, Inc., USA) in front of the LEDs. The samples were positioned on a white Spectralon (Labsphere, USA) and affixed to the white surface using a custom-made frame, with the dyed layers oriented towards the camera. Throughout this paper, the quantity reported and discussed is, for simplicity, the reflectance. It is important to note that this is the reflectance of the film and the white imaging substrate. In terms of the film, it is composed of the true film reflectance and the square of the film transmittance, since light first passes through the film, is subsequently reflected from the substrate, and finally passes the film again before being detected. This process amplifies spectral features, which is desirable for the problem at hand. The simplification is justified in the scope of this paper since only the different spectral shapes of degraded and well-preserved dyes are of interest. The imaging part of the system included an ImSpector V10E imaging spectrograph (Specim, Spectral Imaging Ltd, Finland), a 50 mm lens (Schneider Kreuznach Xenoplan 2.8/50-0902, Jos. Schneider Optische Werke GmbH, Germany) and 5.0MP monochrome CMOS camera (Blackfly S BFS-U3-51S5M-C, Flir Systems Inc., USA). To mitigate specular reflections, a polarizer (Bolder Vision Optik, Inc., USA) was used in front of the objective in a cross-polarized configuration with the LED polarizers. Images were acquired with the resolution of 2048 × 2448 pixels in spectral and spatial dimensions of the spectrograph, respectively. The spectral range considered is from 380 to 780 nm. The field of view in the direction perpendicular to the scanning axis was 73 mm. The system's effective spectral and spatial resolutions were 2.9 nm and 100 µm, respectively, as evaluated by a gas discharge tube and spatial grids used for system calibration. In comparison, RGB images were acquired with a Canon EOS 5D Mark IV camera.

#### Vector quantization algorithm

To gain information needed to restore degraded film, the proposed algorithm relies on two spectral reference images for training: the best preserved of an individual scene and a representative faded frame of the same scene. First, we map the two frames into a space so they may be compared. To do this the preserved *B* and degraded frames *F* are spatially registered pixel-by-pixel. This is conceptually valid as the physical materials in both frames should be made of the same material classes within a given spatial distribution. The only difference is that the degraded frame *B* has a slightly altered chemistry compared to the degraded frame *F*. Therefore, if the spectra of the preserved frame *B* can be successfully clustered, such that the clusters represent the concentration of the photographic dyes, then these clusters should be expected to correlate with the degraded frame *F* intensities. At the core of our algorithm, we are finding the reflectance spectra of the preserved frame that are best related to reflectance spectra of the degraded frame.

To find these mappings, the degraded frame *F* is clustered into K groups to find pixels composed of most similar spectra. We use a vector quantization method analogous to K-means clustering- except without an update step. Once the initial clustering of *F* has been performed, we can predict the response for the preserved frame *B*. The mean of *F* per cluster should be a strong estimate for the composition of the pixels belonging to those clusters. Based on this, one can estimate an image of a “restored” frame by replacing each cluster of pixels in *F* with the mean of *B* response. Thus, the restored frame is estimated from the degraded frame as

1 $$\hat{X}_{restored} = \mathop \sum \limits_{k} \in_{{C_{k} }} \left[ {X_{F} } \right]I_{{C_{k} }}$$

where *k* is the cluster index and *C*_*k*_ is the kth cluster, and $I_{{C_{k} }}$ is the indicator function of cluster *C*_*k*_ .

**Figure 2:**
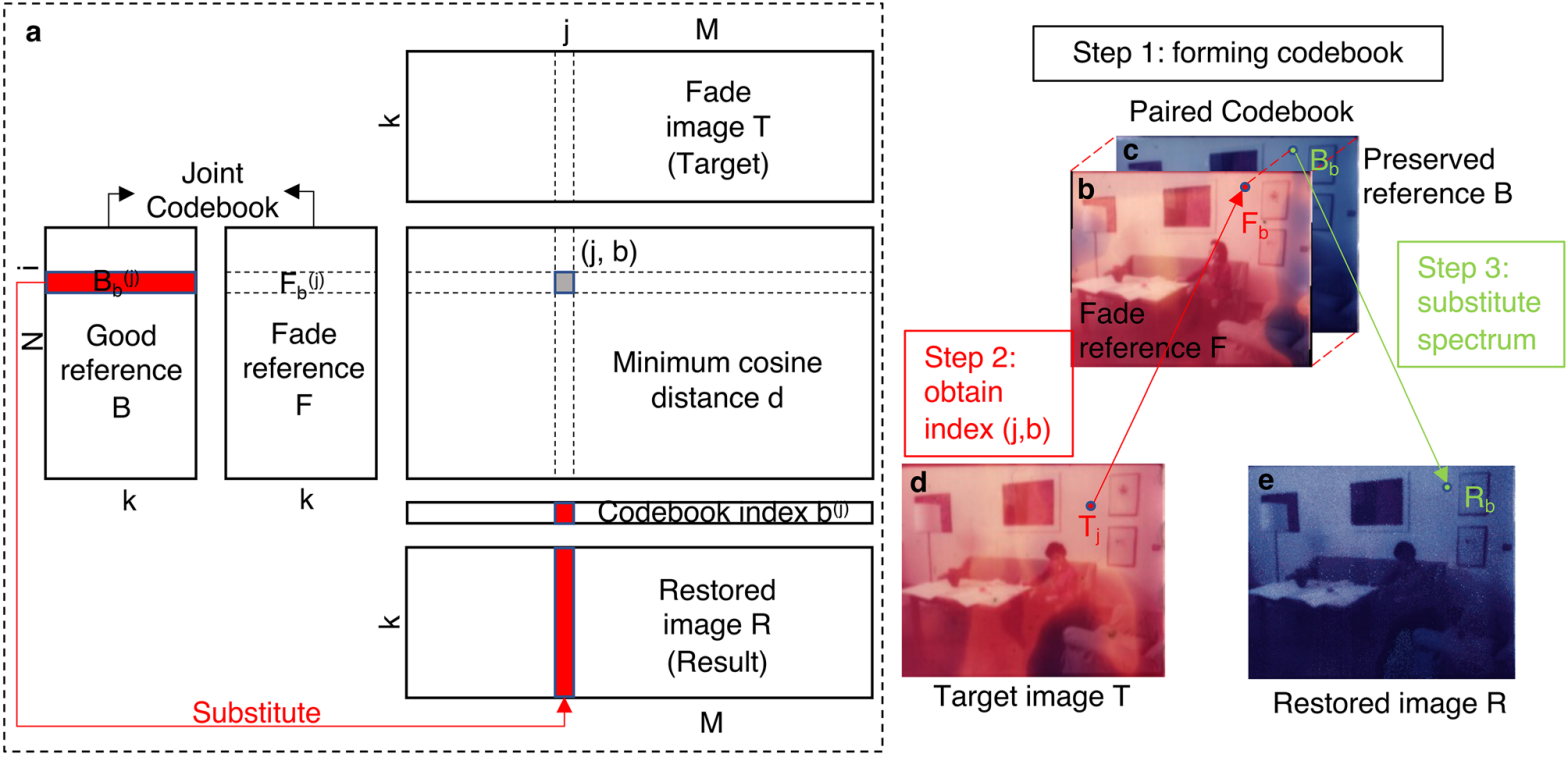
Schematic overview of the vector quantization algorithm. (**a**) k is the spectral wavelength number from the flattened image, N and M represent the total pixel number respectively in the reference image and target image, and (j,b) represents the index of the best representative codeword. (**b**–**e**) RGB representation of fade reference (**b**), good reference (**c**), target frame (**d**) to be restored, and the restoration result (**e**).

The whole process is schematized in Fig. 2 and summarized step-by-step here:

**Step 1** To map the correlations between the degraded and non-degraded states of the film, two references need to be selected: as previously stated, (1) a relatively best-preserved reference frame *B* that serves as the source of "good" spectral signatures and (2) another faded frame *F* of the same scene that provides the degraded spectra. The paired references are then registered pixel-wise using Scale-invariant feature transform (SIFT) correspondence and landmark transformation^30^. Through a pixel-to-pixel correlation, each pixel spectrum on the faded reference *F*_*i*_ has a well-preserved correlated spectrum $B_{i} = a_{i} \cdot F_{i}$, $i = 1, \ldots ,N$ where *a*_*i*_ is the transformation coefficient of each pair of spectra. From this point it is useful to think of these correlated images as a joint codebook C that contains paired information between the unfaded frame and the faded one. Each spectrum thus serves as a codeword, and the paired references form the codebook *C* of *N* paired codewords:

2 $$C = \{ (B_{i} ,F_{i} )|i = 1, \ldots ,N\} .$$

Since the faded frames used in this paper are not artificially simulated (Fig. 1 S1) but actual historical samples representing degradation behaviours, there are subtle content changes from frame-to-frame, such as human figures with slightly adjusted postures. These shifts cause small non-matched pixel regions around the head area that are masked during the following calculation to avoid mismatches.

**Step 2** Looking up the codebook and finding the index. To "translate" a degraded spectrum in the target frame *T* into a best-preserved spectrum, we need to look it up in the codebook first. Therefore, the spectrum *T*_*j*_ at each pixel position in the target needs to be compared with every element throughout the codebook *C* to locate the most representative spectrum *F*_*b*_. The codebook index *b* is learned by calculating the minimum cosine distance *d* between the target spectrum *T*_*j*_ and the reference faded spectrum *F*_*i*_. During this stage, the spectral data cube is unfolded spatially into a flattened image with dimension *Nxk*, where *N* is the total number of pixels and *k* is the wavelength channels. When using RGB images as input data *k* = 3, and for spectral data cubes *k* = 240. After restoration, the unfolded matrix is reformed into the original spatial structure. For each spectrum, *T*_*j*_ in the target cube *T* (Fig. 2d), the cosine distance *d* to every spectrum *F*_*i*_ in faded reference *F* (Fig. 2b) is obtained by

3 $$d\left( {T_{j} ,F_{i} } \right) = \frac{{F_{i} \cdot T_{j} }}{{||F_{i} ||||T_{j} ||}}$$

The resulted distance matrix *d* has the dimension of *NxM*, where *N* is the total pixel numbers in reference and *M* is that in the target. The two images do not necessarily have to be identical in total pixel number and resolution. For the *j*th spectrum in the target, the codeword *F*_*b*_^(*j*)^ that best represents *T*_*j*_ is identified by

4 $$d\left( {T_{j} ,F_{b}^{\left( j \right)} } \right) = \mathop {\min }\limits_{i} d\left( {T_{j} ,F_{i} } \right)$$

which indicates the best match between the target spectrum and the reference spectrum.

**Step 3** Reconstructing the restored image. A target-to-codebook relationship is established via the codebook index $b = \left\{ {b_{1} , b_{2} , \ldots ,b_{j} , \ldots ,\left. {b_{M} } \right\}} \right.$ formed of all elements *F*_*b*_^(*j*)^. Then, exploiting the pixel-to-pixel correlation in the codebook, each spectrum *T*_*j*_ in the target image can be substituted by the corresponding good spectrum *B*_*b*_^(*j*)^ of the best representation *F*_*b*_^(*j*)^ in the codebook. A restored cube R is formed where $R_{j} = B_{b}^{\left( j \right)} = a_{i} \cdot argmin_{i} d\left( {T_{j} ,F_{i} } \right)$ (Fig. 2e). The faded image is reconstructed with the "good" spectral signatures, collected from the good reference, following the calculated index.

VQ has already been applied to many recognition problems associated with language^31^. As an analogy, the principle of this digital restoration strategy is like the process of translating a foreign language. Each faded pixel in a film frame would be an element to be translated into an unfaded pixel. Once the codebook is built, it may be applied to restore any frame with the same degradation characteristics, not limited by size or resolution.

To overcome the limitations of the VQ methods, a multi-codebook was also created to improve the algorithm's accuracy using spectra hand selected from multiple frames. The multi-codebook is one in which the atoms are hand-selected and concatenated from multiple frames.

#### Comparative analysis

Different elaborations were undertaken to compare the application of the novel algorithm on the hyperspectral data. First, RGB acquired images were digitally restored with the professional software *DaVinci Resolve 17* following the instructions available on the website^13^. The RGB data were also tested on our developed algorithm following the same pipelines employed for processing the spectral data (Fig. 2).

## Results and discussions

### Comparative analysis: digital restoration of RGB data

RGB images were acquired with a conventional camera and processed with professional restoration software to evaluate the algorithm's efficiency and reconstruction quality.

For the standard software, the colour correction relies on hand adjusting the parameters until the best possible appearance is obtained according to the operator's skills and aesthetic judgments. Moreover, it is hard to compensate for differential degradation across the frame and achieve a uniform result. Several commercially available restoration software packages were tested using the RGB images (SI paragraph B, fig SI. 2–3).

**Figure 3:**
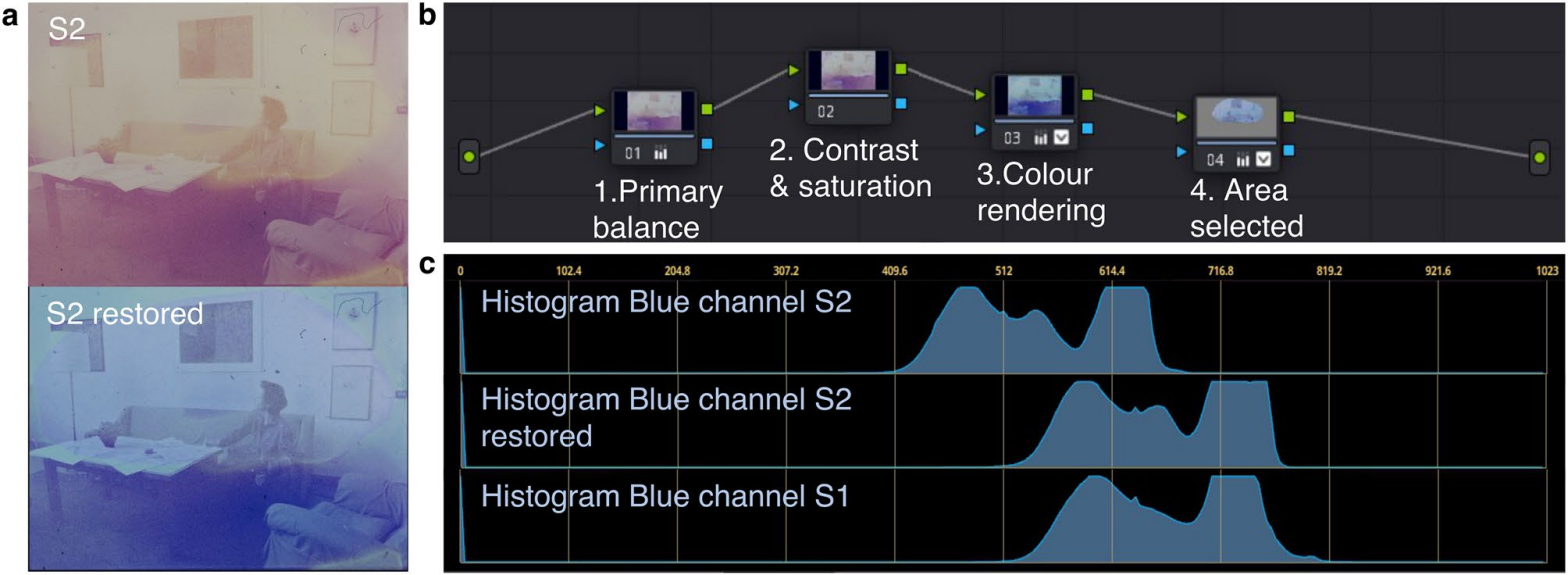
The conventional digital restoration strategy. (**a**) Comparison of RGB images before and after the hand restoration achieved by *DaVinci Resolve 17*. (**b**) Illustration of the processing pipelines. (**c**) Comparison of the histograms before and after restoration with the reference S1.

As an example, Fig. 3a reports the restoration result on frame S2obtained using the *DaVinci Resolve 17*^13^ software as it is one of the commonly used commercial software by film restorers. As shown in Fig. 3b, the primary colour balance was adjusted on the base of selected points on the background (wall areas). Then, two more correction nodes were added to fine-tune the contrast, saturation, hue, tint, temperature, and RGB curves to restore the overall appearance. After those steps, the darker lower half achieved a comparable visual effect to reference S1. At the same time, the more extensively degraded upper part still had a pronounced yellowish hue, which was finally enhanced by selecting those areas and separately adjusting the parameters. The colour appearance of the result (Fig. 3a) appears non-uniform, presenting a colour difference from the reference. It can be confirmed with the histogram graphs, taking the blue channel as an example, shown in Fig. 3c, that the distribution of the colours in the restored image is shifted mainly from the original S2 and now matching with the reference S1.

**Figure 4:**
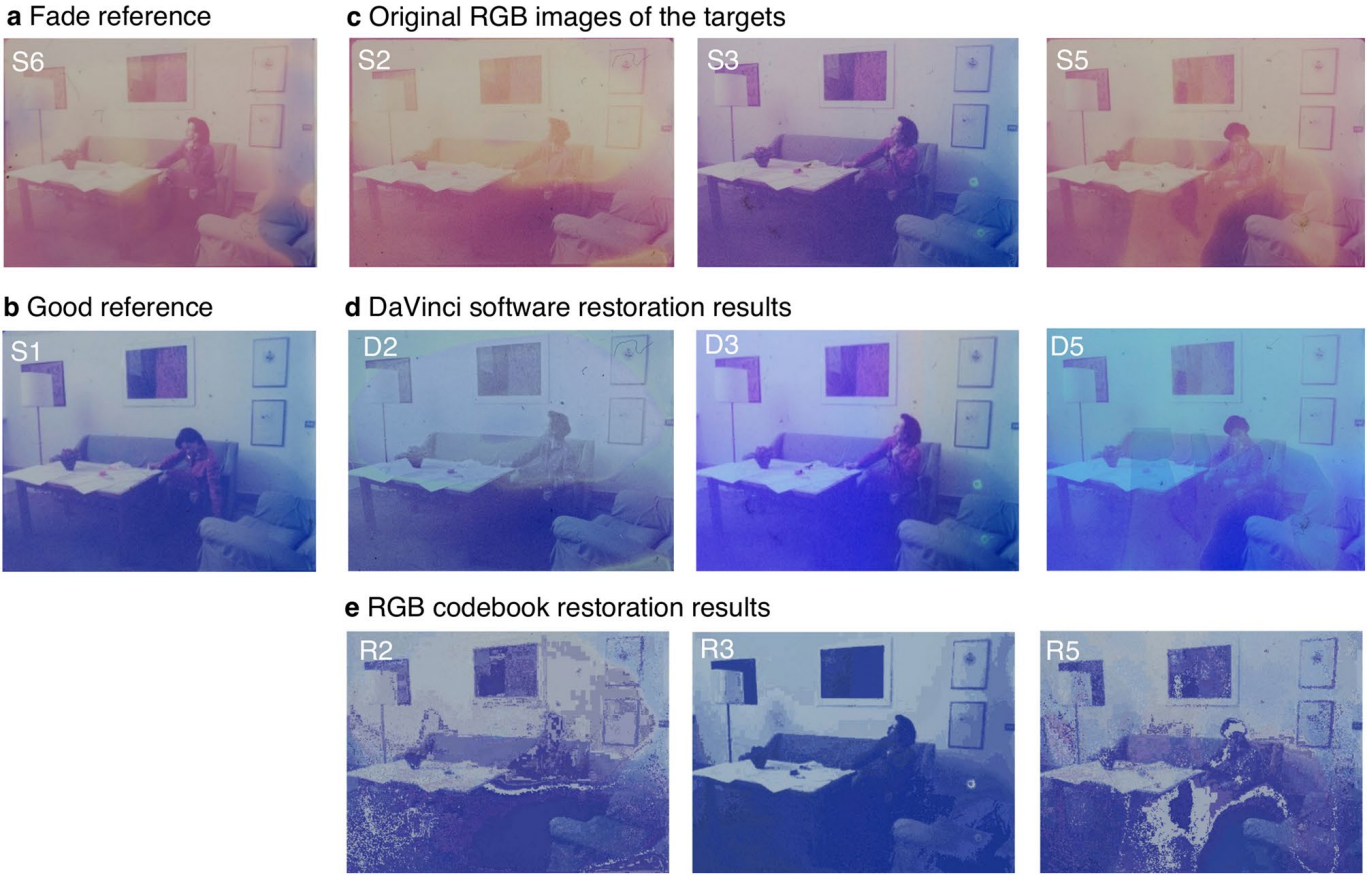
Originals and restoration results obtained via *DaVinci Resolve* software and RGB-codebook approach. (**a**, **b**) Optical RGB images of references S6 and S1. (**c**) Optical RGB images of target frames S2, S3, and S5. (**d**) Manually restored frames D2, D3, and D5 using *DaVinci Resolve* software. (**e**) Restoration results R2, R3, and R5 obtained via RGB codebook approach.

The overall balance of the RGB colours is also adjusted closer to the reference, but the restored image still presents inconsistencies that are hard to correct completely. The same procedure was repeated also on frames S3 and S5 (Fig. 4d). The presenting the same problems previously described and leading to a not uniform appearance of the restored frames Thus, the results achieved by conventional practice are limited by the restorer's subjective choice, personal taste, and proficiency. Moreover, the processing of one single frame takes up to 30 min and for historical films with inhomogeneous degradations, the fine-tuning of parameters is inevitable from frame to frame.

**Figure 5:**
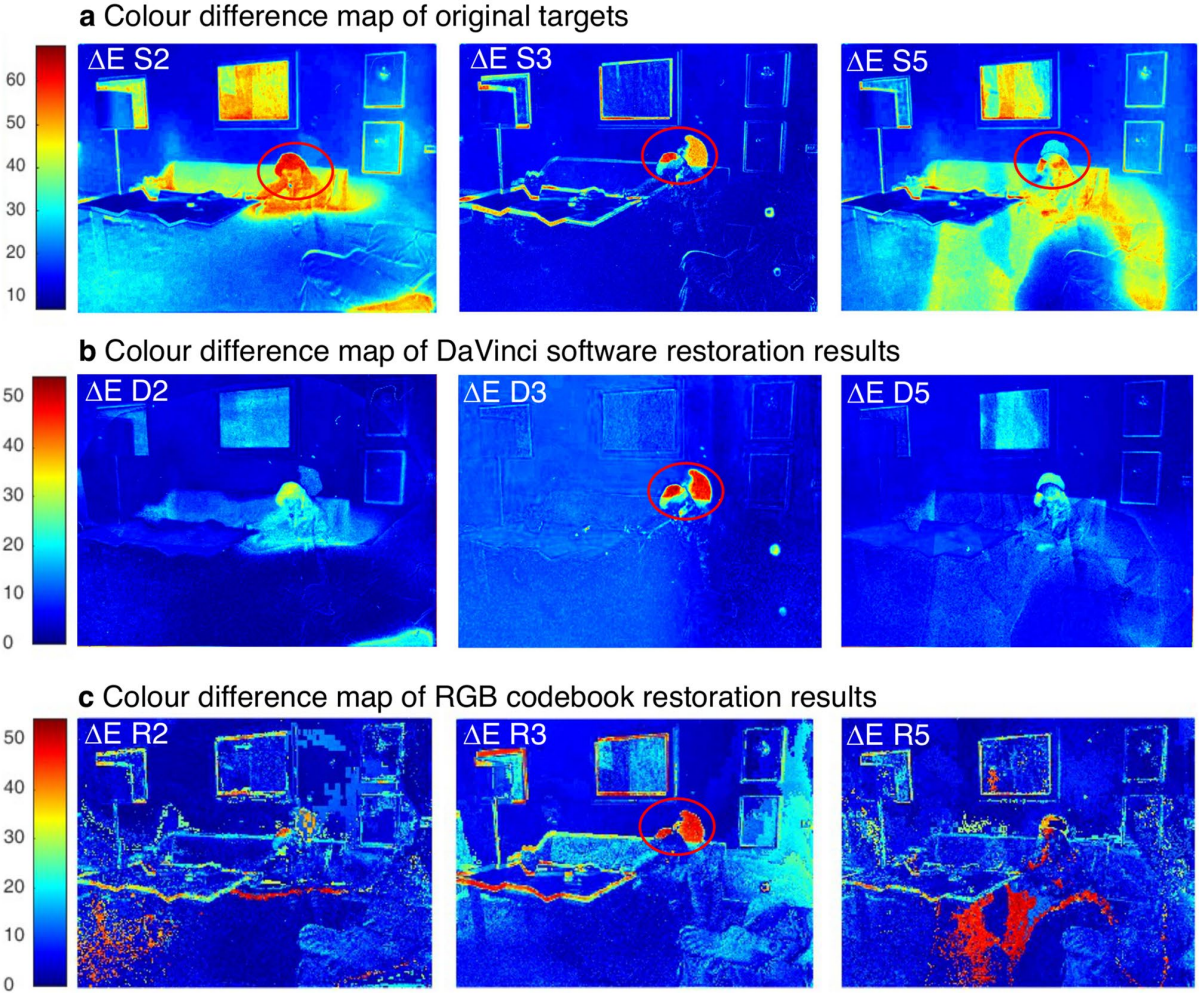
Evaluation of the results obtained via RGB images based approaches. (**a**) Colour difference (ΔE) map of the original target frames S2, S3, and S5 compared to the reference S1. (**b**) Colour difference (ΔE) map of the restoration results D2, D3 and D5 using *DaVinci* software. (**c**) Colour difference (ΔE) map of the restoration results R2, R3 and R5 obtained via RGB triplet codebook approach.

The VQ algorithm was applied to RGB data to evaluate the advantages of processing with hyperspectral data. In particular, a codebook was created using S1 and S6 as paired references (Fig. 4a, b). The wavelength channel has a dimension *k* = 3. Then the digital restoration was achieved on target images S2, S3, and S5 by the vector quantization method proposed above, finding the best representative codewords in the fade reference S6 and then substituting each pixel with the corresponding good one. The results are reported in Fig. 4e. A simple evaluation of the restoration performance is based on calculating the colour difference ΔE between the obtained results *R* and reference *S1* per pixel through Euclidean distance:

5 $$\Delta {\text{E }} = \sqrt {\left( {r_{R} - r_{S1} } \right)^{2} + \left( {g_{R} - g_{S1} } \right)^{2} + \left( {b_{R} - b_{S1} } \right)^{2} }$$

where *r*, *g*, and *b* represent RGB channel values. The resulting greyscale matrices ΔE are shown as colour maps marked with the colour scale (Fig. 5), where higher ΔE values are marked in warm colour, and smaller ΔE values are drawn in blue. As references for the initial level of colour difference, the original colour difference maps (Fig. 5a) are also obtained by calculating ΔE between the good reference S1 and each target image before restoration. Areas highlighted in red and yellow indicate more significant colour differences with respect to the reference, thus more degraded than the areas marked in blue. However, it is worth mentioning that the rise of ΔE in contours is due to the non-perfect alignment between the RGB image pairs, as is evident in Fig. 5a S3. Then a quantitative estimation of the overall performance *dE* for each image is obtained by averaging the colour difference ΔE for all pixels:

6 $${\text{dE}} = \frac{1}{M}\left( {\mathop \sum \limits_{i = 0}^{M} \Delta {\text{E}}_{i} } \right)$$

and listed in Table 1.

**Table 1:** Calculation results of averaged colour difference *dE* and peak signal-to-noise (PSNR) level of frames S2, S3, and S5.

Frame no	Original dE	Colour difference (dE)				PSNR (in dB)			
		*DaVinci* Restored	RGB codebook	Simple codebook	Multi codebook	*DaVinci* Restored	RGB codebook	Simple codebook	Multi codebook
S2	24.3901	7.9507	7.3894	4.7016	3.8401	22.1350	21.1825	25.3592	25.8963
S3	6.3129	7.7945	9.0021	6.2975	4.1670	19.4800	19.3644	22.3881	25.1200
S5	22.6927	8.8564	8.7019	6.7695	4.3850	19.6584	19.6892	22.5719	25.3910

Observing the restored R2 and R5 images in Fig. 4e, it can be highlighted that even though the basic structure of the images is maintained, many pixels were mismatched, especially in the most extensively degraded areas. This is confirmed by the colour difference map shown in Fig. 5c. For frames S2 and S5, even though the overall colour differences have decreased, from 24.3901 before restoration to 7.3894 after restoration in S2 and from 22.6927 before restoration to 8.7019 after restoration in S5 (as reported in Table 1), there are several pixels mismatched, mainly in the most extensively degraded areas (Fig. 5c).

As a comparison, the results obtained by *DaVinci* manual restoration (Fig. 4d) are more uniform in overall appearance without the irregular mismatching pixels (Fig. 5b). However, the average colour difference for conventional software method is slightly higher than what obtained using the RGB codebook results, where for results D2 dE = 7.9507 (dE_R2_ = 7.3894, Table 1) and for D5 dE = 8.8564 (dE_R5_ = 8.7019, Table 1), resulting from the overall shifts in colour. The accurate colour representation is quite challenging using conventional restoration software.

On the other hand, the original frame S3 (Fig. 4c S3), which is much less degraded than samples S2 and S5, is more uniform in colour and has more negligible colour difference with respect to reference S1 (ΔE = 6.3129, Table 1). However, since the degradation characteristics are very different from those included in the fade reference (Fig. 4a), the restoration result (Fig. 5c R3) is less accurate with a higher ΔE value (9.0021, Table 1), especially in the wall painting areas and dark part on the right of the image. For restoration result D3 obtained with *DaVinci Resolve* (Fig. 4d), the colour difference ΔE is also elevated to 7.7945, though still the smallest among all three frames. In this case of the RGB codebook, the matching accuracy is primarily limited by the short spectral vector formed from the RGB triplet values as source data. Nevertheless, given the overall evaluation and quick processing time (seconds) compared to hand restoration, the VQ technique still performs promising.

### Vector quantization of hyperspectral data: simple codebook approach

**Figure 6:**
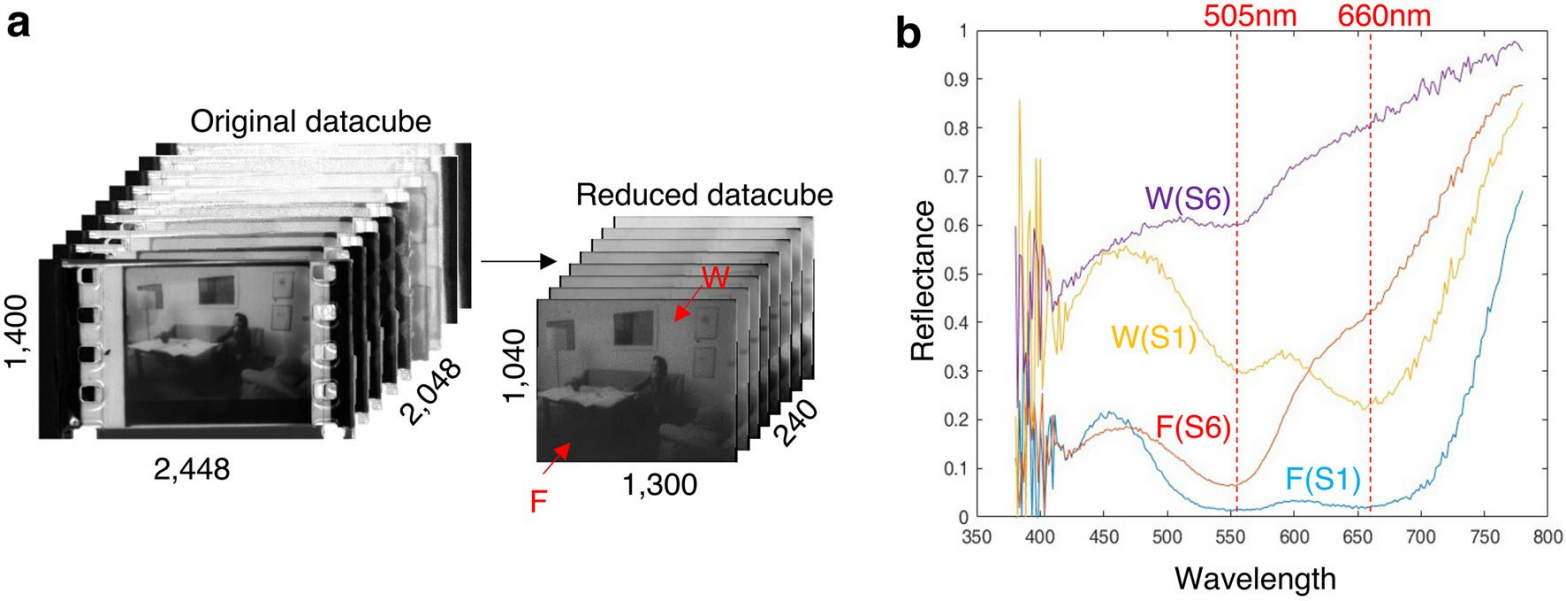
Datacube processing and selected spectra. (**a**) Greyscale illustration of original datacube before and after initial processing. (**b**) Comparison of spectrum extracted from the same x and y coordinates on S1 and S6, respectively.

The proposed codebook method was performed on the spectral data obtained with the VNIR hyperspectral camera^29^. The collected data cube with dimensions of 2448 × 1400 spatial pixels × 2048 wavelengths was cropped and binned to a spectral range limited to 380 nm to 780 nm (240 channels separated by approximately 1.8 nm) and spatial dimensions of 1300 × 1040 pixels as described in the section above and shown in Fig. 6a. The high-resolution three-dimensional data cube provides richer than the RGB image since it contains spectral features associated with dye molecule deterioration. Reflectance values are affected by two factors. First, every pixel on the film may contain a different level of dye density due to variation in image content. For instance, the brighter wall (pixel W in Fig. 6) has a lower dye density than the dark floor (pixel F in Fig. 6), thus a higher reflectance value. Secondly, less light is absorbed as the fading becomes more severe, contributing to the more intense reflectance values observed.

Two pairs of spectra, one from the wall (pixel W) and the other from the floor (pixel F), were extracted from corresponding pixels in the better-preserved S1 and the degraded S6. The comparison of spectra (Fig. 6b) shows that the wall pixel on deteriorated film W(S6) has a significantly higher reflectance across the spectral range than sample S1. Furthermore, the deteriorated film F(S6) drastically loses the absorption band around 660 nm compared to the best-preserved sample F(S1). This feature is related to the degradation of cyan dyes that lead to the overall purplish hue of the faded film. Those subtle spectra variations serve as the fingerprints for looking up the most representative spectrum in the codebook.

**Figure 7:**
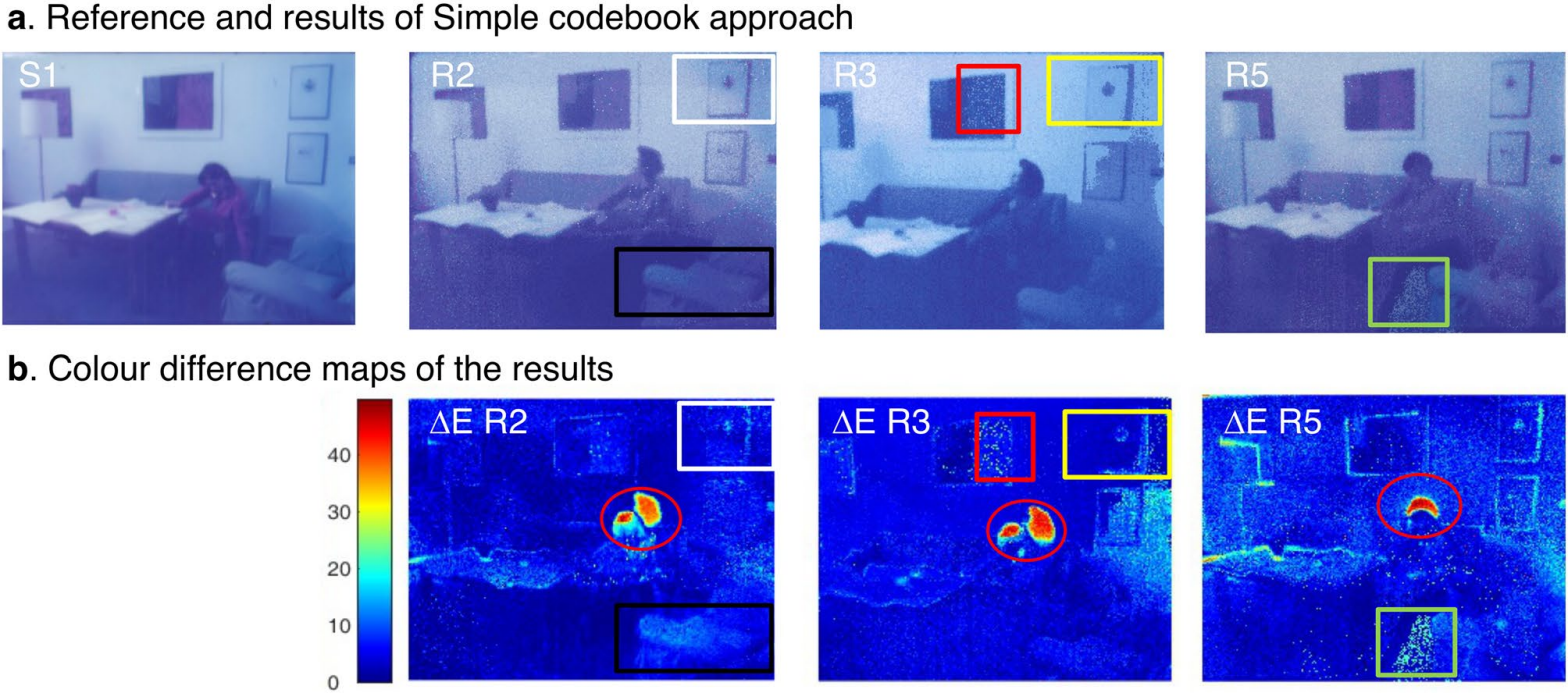
Results and evaluation of simple codebook approach. (**a**) Digital restoration outcomes R2, R3, and R5 were obtained via a codebook approach visualized in RGB format. (**b**) Colour difference map of the above restoration results in a simple codebook approach.

For convenience, the restored data cubes are transformed into RGB space, as shown in Fig. 7a, using wavelength weighting methods described in supplementary information section C. It can be observed that the restoration results (Fig. 7a) contain many fewer mismatched pixels than those tested using RGB triplet values (Fig. 4e), with overall lower ΔE values in all three frames (Table 1). Employing the vector quantization algorithm, however, the accuracy of the restoration is still dependent on the selected reference spectra. If a spectrum on the target image is not contained in the reference, the perfect match cannot be found, and a rise in noise level and shift in hue would be expected. As already discussed in RGB codebook results (Fig. 5c R3), frame S3 restored using a simple spectral codebook (Fig. 7a R3) still presents unwanted bluish hues in the background and mismatched shade on the right (marked in the yellow square), though much more limited than what obtained with the RGB approach. The improvement of colour difference (from 9.0021 in RGB codebook to 6.2975 in simple codebook approach, Table 1) could also be observed in the colour difference map (Fig. 7b R3), where pixels with high ΔE value disappeared in most of the areas and decreased in intensity as compared to Fig. 5c R3. On the other hand, frames S2 and S5 that have similar degradation features with reference S6 achieved better restoration accuracy, with significant mismatches corrected.

### Vector quantization of spectral data: multiple codebook approach

**Figure 8:**
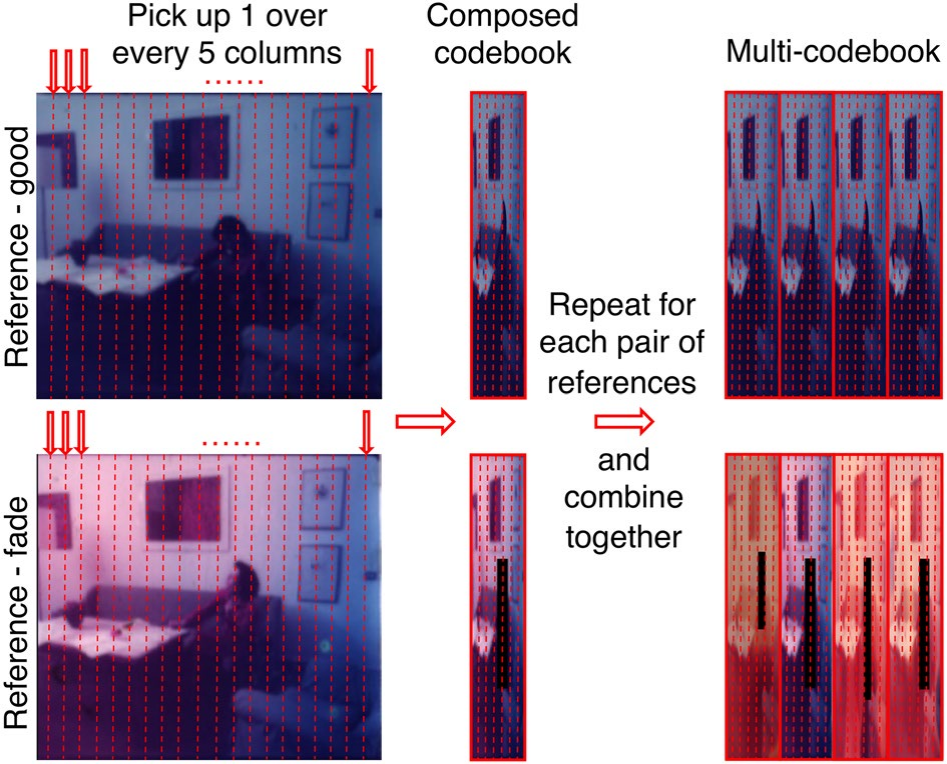
Schematic overview of constructing multi-codebook, using spectra hand-selected and combined from multiple samples S2, S3, S5, and S6.

To improve the representativeness of the spectra and the algorithm's applicability, a multi-codebook was created using spectra hand selected from multiple frames. Since the data cube is collected with a high spatial resolution, adjacent pixels are mostly similar and highly repeating. Taking every pixel into the codebook would produce a large vector, resulting in a high computational load. As illustrated in Fig. 8, a selection of pixels from S2, S3, S5, and S6 was employed to create the multi-codebook. S1 was used as an unfaded reference to each faded reference frame, and the pixel-to-pixel correlation was maintained between the pair of references. One out of every five columns for each frame are kept reducing over-sampling, preserving the total variance and representativeness of the spectral features. Then, the four compressed reference cubes are combined to form a new multi-codebook reference containing spectral information from all instances. The multi-codebook was tested for each frame via the same vector quantization and index substituting process.

**Figure 9:**
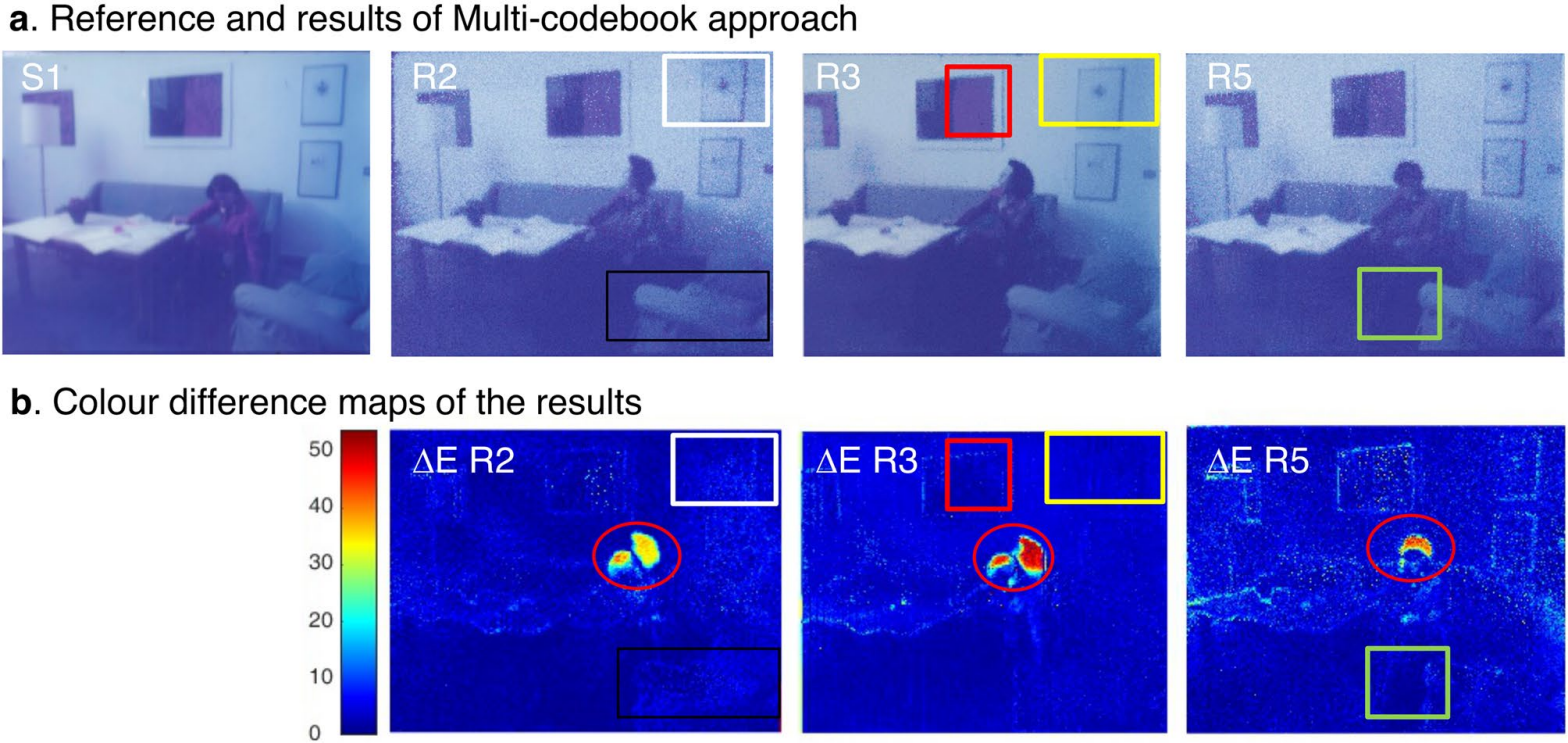
Results and evaluation of the multi-codebook approach. (**a**) Digital restoration outcomes R2, R3, and R5 were obtained via a multi-codebook approach visualized in RGB format. (**b**) Colour difference map of the above restoration results in a simple codebook approach.

The results of the digital unfading via the multi-codebook approach are reported as RGB images in Fig. 9a.

Restoration using the three-codebook approach (Figs. 4, 7, 9) achieved the most satisfactory restoration accuracy with the lowest ΔE values (Table 1). From the colour difference maps (Fig. 9b), it could also be confirmed that the unevenly degraded pixels in the original frames (Fig. 5a) are recovered, achieving a uniform appearance, and the mismatches occurred in RGB codebook, and simple codebook approaches are now corrected. For frame S3, the noticeable shift in hue and the mismatching on the right (highlighted in yellow square in Fig. 7 R3) disappeared when the more representative multi-codebook was used (in Fig. 9 R3). Furthermore, the noise of the painting hanging on the wall (highlighted in red square in Fig. 7 R3) is removed, and the colour is restored closer to the reference when using a multi-codebook (Fig. 9 R3). For frame S2, the lighter desk and the brightness and contrast of the sofa (highlighted in a black square in Fig. 7 R2) are corrected and improved when using multi-codebook (Fig. 9 R2) and are now closer to the reference image. The restoration results obtained through simple and multiple codebooks are comparable for sample S5, which shares the most similar degradation features with reference S6 (Fig. 4a). At the same time, a small noisy area around the sofa (highlighted in the green square in Fig. 7 R5) is successfully corrected with a multi-codebook (in Fig. 9 R5). The pixels of significant colour difference concentrated in the head area (marked in the red circle in Fig. 9b) are not restoration errors, as could be checked with Fig. 9a, but due to the non-overlapping of the human figure (as already noticed in Fig. 5a) in the target frames with the reference S1.

### Comparison of the different restoration methods

To better evaluate the quality of the reconstruction, peak signal-to-noise ratio is also estimated for all the results (Table 1). The Root Mean Square Error (*RMSE*) between the restoration result *R* and the reference *B* is first calculated. Then, the PSNR value (in dB) of the reconstructed image *R* is estimated by:

7 $$PSNR = 20\log_{10} \left( {\frac{{MAX_{B} }}{RMSE}} \right)$$

where *MAX*_*B*_ is the maximum signal value in the referential ground truth image B. This measurement is conducted both on the restoration results obtained from the conventional restoration software and on the transformed RGB representation from the spectral results achieved via codebook approaches.. The image format is double decibel in our process, so the *MAX*_*B*_ here is 1. Following the definition of PSNR, the higher the value, the better quality of the degraded image has been reconstructed.

Observing the results in Table 1, the results achieved with RGB triplet codebook are comparable with those obtained with the conventional restoration software *DaVinci Resolve 17*, while the spectral codebook approaches tend to have significantly higher PSNR levels, indicating a better reconstruction quality. The general tendency of increasing PSNR level when including more elements in the codebook is also observed. The multi-codebook outperformed all other tested methods, obtaining the highest PSNR level while achieving the best colour difference metrics. Even though the image quality is improved the most with the multi-codebook approach, there is still noticeable noise in the reconstruction results. This noise is presumably connected to the still over-abundant sampling rate when constructing the codebook. Other data reduction methods, such as clustering and segmentation techniques to remove the non-correlated elements and only keep the representative centroids of each cluster, may help in further reducing the noise.

In conclusion, this work demonstrates that imaging spectroscopy combined with digital unfading machine learning technique successfully restores historic motion pictures with inhomogeneous fading, obtaining a result which is hard to achieve with conventional method. Our vector quantization method has been positively tested with a pipeline of data processing techniques to restore faded cinematic film, mainly because of the high-resolution spectral features that capture the minimal but essential differences among pixels. The constructed multi-codebook could be applied to restore deteriorated images of the same type. Moreover, the spectra bank collected in the codebook could be further expanded, including different types of samples and degradation effects, to apply to a more extensive range of damaged films. This may allow the automatic restoration of several images of the same movies simultaneously.

A drawback of the method could be represented by the large size of the high-resolution spectral data files, which may result in long computational time processing. This study overcame the problem by down sampling the images before applying the algorithm.

As future perspective, the method can be further improved by applying the clustering techniques, such as k-means clustering, to include only the centroids of each obtained cluster in the spectra bank. Forming a more compact multi-codebook, the computational load will be further lightened, and the noise level is expected to be reduced. The proposed method could also be transformed into a robust dictionary learning program to complete the tasks.

## Supplementary Information

Supplementary Information.

## Data Availability

Data are available with the permission of the University of Bologna. Due to the large size of the spectral data involved, raw and processed data can be made available upon reasonable request. Please contact s.prati@unibo.it.

## Code availability

The corresponding author can provide MATLAB codes used in this work upon reasonable request.
