# Supplementary material for: Digital restoration of colour cinematic films using imaging spectroscopy and machine learning
Source: Sci Rep. 2022 Dec 20;12:21982. doi: 10.1038/s41598-022-25248-5 (PMC9767916; doi:10.1038/s41598-022-25248-5)
Supplement: Supplementary file 1 — Supplementary Information. [file 41598_2022_25248_MOESM1_ESM.pdf]

Digital restoration of colour cinematic films using imaging spectroscopy and machine learning-Supplementary information

L. Liu[1], E. Catelli[2], A. Katsaggelos[3], G. Sciutto[2], R. Mazzeo[2], M. Milanic[4,5], J. Stergar[4,5], S.Prati[2]*, M. Walton[6]*

1. Department of Informatics-Science and Engineering, University of Bologna, Mura Anteo Zamboni, 7-Bologna, Italy

2. Department of Chemistry "G. Ciamician", University of Bologna, Via Guaccimanni, 42–48121, Ravenna, Italy

3. Department of Electrical Engineering and Computer Science, Northwestern University, 3270 Evanston, IL, USA

4. Faculty of Mathematics and Physics, University of Ljubljana, Jadranska cesta 19–1000, Ljubljana, Slovenia

5. "Jožef Stefan" Institute, Jamova cesta 39-1000, Ljubljana, Slovenia

6. Department of Conservation and Research, M+ Museum, 38 Museum Drive, West Kowloon Cultural District, Hong Kong

***: corresponding authors: s.prati@unibo.it; marc.walton@mplus.org.hk**

A. Samples' description

In this work, miscellaneous positive prints were provided by L'immagine Ritrovata Film Restoration Laboratory in Bologna, taken from a historical film fragments. Each frame is measured to have physical dimension of 34 mm x 19 mm (± 0.5 mm). The FUJI N4 edge mark is found on the perforation, identifying the manufacture as FUJI film (fig. SI1). In addition, "N4" indicates the support material to be cellulose acetate, and the code "86-AJ" were used to indicate the manufacture time which correspond to April-June 1986. The Fuji film set has thickness around 130um, excluding the protective tapes attached on the surface. The emulsion layer has trichromatic structure with thickness around 15um, in dye sequence of yellow, magenta, and cyan. The dyes located in different layer is degraded in separate rate through time[1].

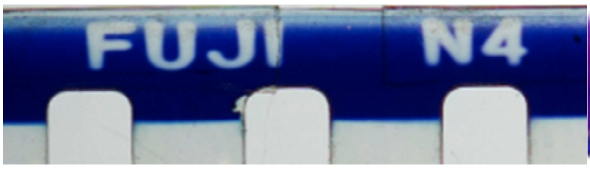

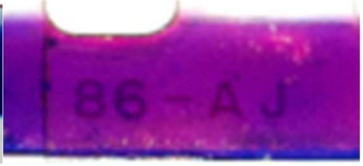

Figure SI1. The edge mark "FUJI N4" and "86-AJ" found on the perforation of film sample S4 (left) and S5 (right).

B. Common restoration practice

As a comparison to our approach, we tested several commercially available restoration software to digital restore the colour of our faded films. A first attempt was made using *Photoworks Photo Editor 2021* [2] (Fig. SI2). Their retouch tool could effectively remove structured defects, such as dirt and cracks, and restore the image to a clean state as done by chemical cleaning. The colour restoration, however, depends on the hand adjustment, which is time-consuming and highly subjective. It is hard to separately treat the more degraded area and remove those stains, bringing

the entire image to a uniform appearance. For unskilled non-professions like us, the best we can achieve still presents large colour difference from the reference.

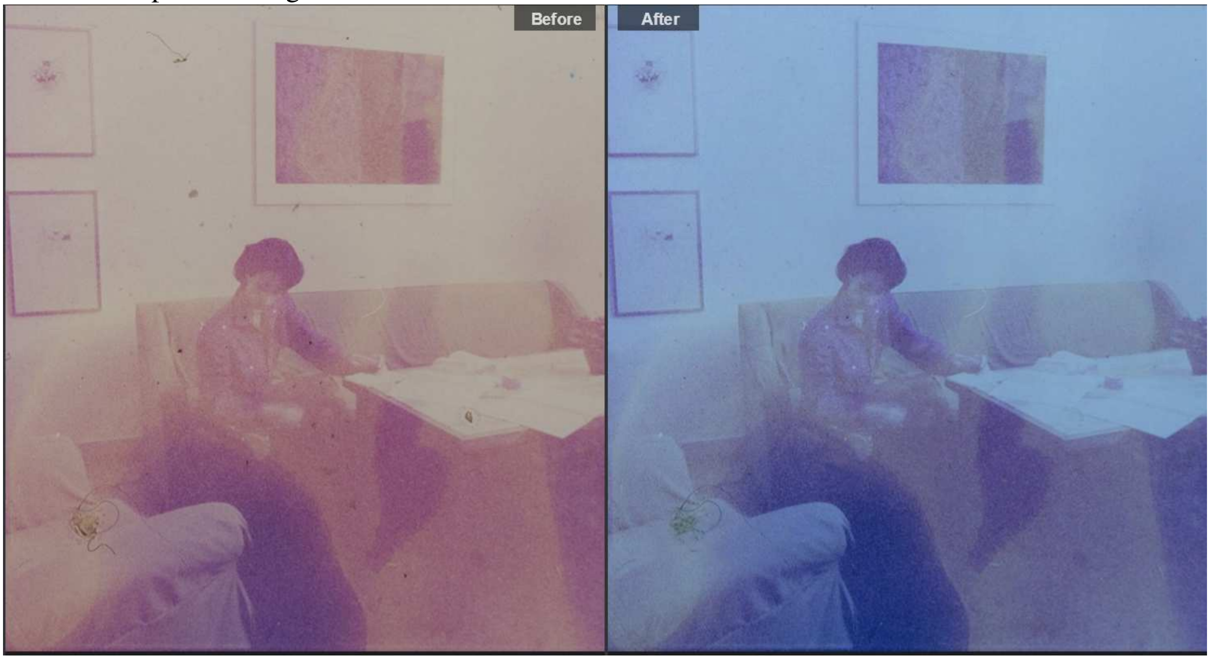

Figure SI2. The RGB image before and after the retouch using *Photoworks software*.

Other more automatic restoration softwares are also tested (fig.SI3). The automatic fade correction enabled by *Corel Paintshop Pro*[3], noted to base on artificial intelligence technology, consider the fading to be homogenous on the image not only spatially, but also among different type of dyes. The enhanced image is even more unbalanced, and the lost information of the already degraded dyes could not be recovered through this type of practice. On the other hand, the *SoftOrbits Photo Retoucher*[4], designed specifically for restoring old photos, is equipped with automatic re-colorization function. However, this program is developed based on black and white photo. The restoration result appears yellowish and lost completely the unique colour characteristics.

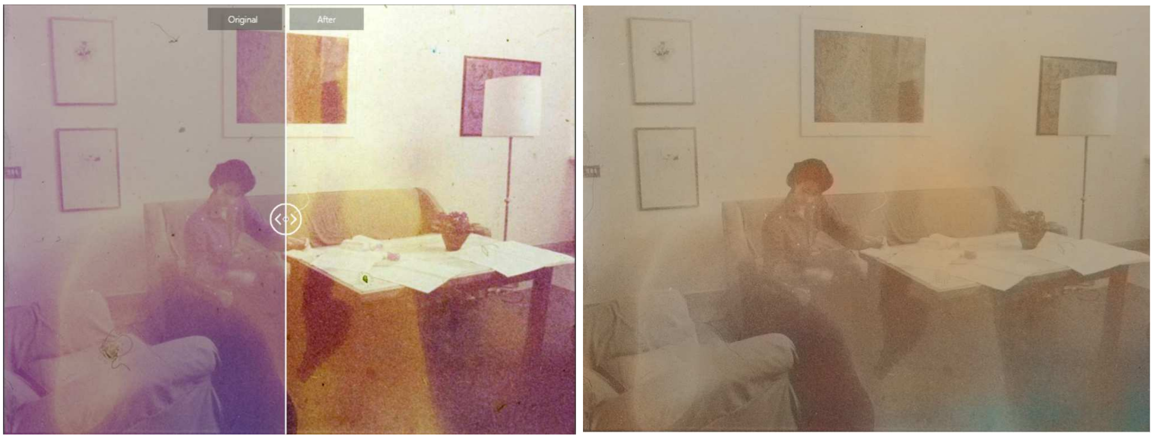

Figure SI3. The RGB image before and after the automatic fade correction using *Corel Paintshop Pro* (left) and re-colorized by *SoftOrbits Photo Retoucher* (right).

C. Transformation from HSI to RGB

The transformation from hyperspectral data to RGB values is performed through a inter stage of CIE XYZ colour space[5]. The CIE 1931 RGB colour space and CIE 1931 XYZ colour space created by the International Commission on Illumination (CIE) is the numerical representation of the

relationship between light spectrum and the chromatic response of the human vision [5]. Using those CIE data as standard (measured every 5 nm, 2 degree), the CIE XYZ Tristimulus values could be calculated for the input visible spectra. Finally, the tristimulus values, which is device-invariant representation of colour, is converted from XYZ colour space to sRGB colour space through a strictly linear transformation:

```
M = [3.2406 -1.5372 -0.4986
     -0.9689 1.8758 0.0414
     0.0557 -0.2040 1.0570];
sRGB = (M*XYZ')';
```

References

1. Egerton, G. S. & Morgan, A. G. The Photochemistry of Dyes II—Some Aspects of the Fading Process. *J. Soc. Dye. Colour.* **86**, 242–249 (1970).
2. How to Restore Old Photos Without Photoshop. *Photoworks Photo Editor* https://photo-works.net/how-to-restore-old-photos.php (2021).
3. How To Restore Photos. *Corel Paintshop Pro* https://www.paintshoppro.com/en/tips/corrections/restore-photos/ (2021).
4. Old Photo Restoration Software. *SoftOrbits Photo Retoucher* https://www.softorbits.net/old-photo-restoration-software/ (2021).
5. Conde, J. *et al.* CIE-XYZ fitting by multispectral images and mean square error minimization with a linear interpolation function. *Rev. Mex. Física* **50**, 601–0 (2004).
